# A Descriptive Study of the Pattern of Refractive Errors, Amblyopia Burden, and Effective Refractive Error Coverage Among Children Attending a Tertiary Hospital in Northern Sri Lanka

**DOI:** 10.7759/cureus.114006

**Published:** 2026-08-05

**Authors:** Malaravan Muthusamy, Thurga Jeyaratnam, Kumanan Thirunavukarasu, Subaschandren Kumaran, Powsiga Uruthirakumar, Jathusha Kanmanirajah, Aruljenani Kumutharanjan

**Affiliations:** 1 Department of Ophthalmology, Teaching Hospital Jaffna, Jaffna, LKA; 2 Center for Digital Epidemiology, Faculty of Medicine, University of Jaffna, Jaffna, LKA; 3 Medicine, Faculty of Medicine, University of Jaffna, Jaffna, LKA; 4 Community and Family Medicine, Faculty of Medicine, University of Jaffna, Jaffna, LKA

**Keywords:** astigmatism, effective spectacles coverage, low- and middle-income countries, myopia, refractive error, school children

## Abstract

Background: Visual impairment affects children's learning, development, and quality of life. Refractive errors (REs) are the most prevalent form of VI among ocular conditions, especially in children. They often begin in childhood and can progress significantly if left uncorrected, leading to complications such as amblyopia or long-term visual disability. Thus, this study was conducted to describe the patterns of REs, examine demographic and behavioral characteristics, and assess the burden of amblyopia and effective RE coverage among schoolchildren with REs attending the Ophthalmology Unit of Teaching Hospital Jaffna, Sri Lanka.

Methodology: This hospital-based prospective cross-sectional study was conducted at the Ophthalmology Unit of Teaching Hospital Jaffna. Data were collected over two months among schoolchildren aged 6-18 years with REs. A total of 253 participants were recruited using systematic random sampling. Data on demographic factors, family history, outdoor activities, screen exposure, spectacle use, visual acuity, and RE patterns were collected using an interviewer-administered questionnaire and medical records from routine eye examinations. Visual assessments and refraction were performed according to standard ophthalmic procedures. Data were analyzed using IBM SPSS Statistics version 25.0 (IBM Corp., Armonk, NY, USA) with descriptive statistics.

Results: A total of 253 schoolchildren with REs were included, with a mean age of 11.7±3.6 years; 138 (54.5%) were female. Astigmatism was the most common RE (118, 46.6%), followed by myopic astigmatism (66, 26.1%) and myopia (58, 22.9%). A family history of RE was present in 33.2% (n=84) of participants. The effective RE coverage was 18.6%. Among children previously prescribed spectacles, only 47 (33.3%) were current users, with lack of information (58, 61.7%) and affordability (20, 21.3%) being the main barriers to spectacle use. Amblyopia was present in 152 (60.1%) participants and was most common among children with hyperopic astigmatism (5, 100%) and hyperopia (5, 83.3%).

Discussion: Astigmatism was the most common RE among children attending a tertiary eye care center in Northern Sri Lanka, followed by myopic astigmatism and myopia. A high burden of amblyopia and very low effective RE coverage were observed, indicating inadequate utilization of refractive services despite spectacle prescriptions; the high amblyopia burden likely reflects the tertiary referral-based study population rather than the general community. Lack of information and affordability were the main barriers to spectacle use. These findings highlight the need to strengthen early vision screening, improve awareness of spectacle compliance, and enhance access to affordable eye care services for children in Northern Sri Lanka.

Conclusion: As the first hospital-based evidence of its kind from Northern Sri Lanka, these findings provide a starting point for local eye care planning while highlighting the broader need for community-based research to establish the true population burden. Strengthening screening pathways, support for spectacle compliance, and the affordability of eye care should be prioritized as immediate steps toward reducing preventable childhood VI in this region.

## Introduction

Vision is one of the senses essential for human development throughout life. Visual impairment (VI) affects children's learning, development, and quality of life. Refractive errors (REs) are the most prevalent form of VI among all ocular conditions, especially in children. REs are optical anomalies that cause light rays to focus improperly on the retina, resulting in blurred or distorted vision. The main types of REs include myopia (nearsightedness), hyperopia (farsightedness), and astigmatism [1]. These errors often begin during childhood and can progress significantly if left uncorrected, leading to complications such as amblyopia or even long-term visual disability. However, they can be corrected with spectacles, and timely diagnosis and treatment can prevent the adverse effects of uncorrected refractive error (URE).

Globally, the World Health Organization (WHO) estimates that 12.8 million children are affected by URE, with a prevalence of 0.96%, the highest reported in urban and highly developed areas of Southeast Asia and China [2]. In low- and middle-income countries, including Sri Lanka, REs are a growing public health concern, particularly because of lifestyle changes and increased screen time that contribute to rising rates of myopia among children [3].

In Sri Lanka, REs remain a leading cause of preventable childhood VI. Although school eye screening programs have been implemented nationally, many challenges persist in ensuring equitable access to refraction services, particularly in resource-constrained and post-conflict regions such as Northern Sri Lanka. Most existing evidence in Sri Lanka comes from school-based surveys, whereas hospital-based data, particularly from a tertiary care teaching hospital, remain limited. There are few studies reporting prevalence estimates among schoolchildren, and evidence describing the patterns of REs among children presenting for care at a tertiary hospital in this region is scarce. Data on the distribution of RE patterns, severity, and demographic associations among children receiving hospital-based services are crucial for informing regional eye health strategies, optimizing service delivery, and strengthening referral pathways between schools and tertiary hospitals. To the best of our knowledge, this is the first hospital-based study in Sri Lanka to assess the patterns of REs among schoolchildren.

Therefore, this hospital-based prospective cross-sectional study was conducted to describe the patterns of REs, examine demographic and behavioral characteristics, and assess the burden of amblyopia among schoolchildren with REs attending the Ophthalmology Unit of Teaching Hospital Jaffna, Sri Lanka. The findings from this study provide critical insights toward the goal of eliminating preventable VI and promoting optimal visual health among Sri Lankan schoolchildren.

## Materials and methods

Study design and setting

This hospital-based prospective cross-sectional study was conducted from April 2025 to December 2025 at the Ophthalmology Unit of Teaching Hospital Jaffna. The Ophthalmology Unit of Teaching Hospital Jaffna is the primary tertiary referral center for eye care in the Northern Province of Sri Lanka. The unit receives children from both rural and urban areas, providing a diverse patient population. The study was approved by the Ethics Committee of the Faculty of Medicine, University of Jaffna (Reference No. J/ERC/25/173/NDR/0348), and the Director of Teaching Hospital Jaffna granted permission to access participants.

Study population

The study population comprised schoolchildren aged 6-18 years who were enrolled in regular schooling, spoke Tamil, and attended the Ophthalmology Unit of Teaching Hospital Jaffna for eye care during the study period. Only those diagnosed with REs were included. Written informed consent was obtained from each participant's parent or legal guardian. Children aged 12-18 years provided written assent, whereas those younger than 12 years provided oral assent. Eligible participants were selected according to predefined inclusion and exclusion criteria. The criteria used for participant selection are presented in Table 1.

**Table 1:** Inclusion and exclusion criteria for study participants

Category	Criterion
Inclusion	Children aged 6-18 years
	Enrolled in regular schooling
	Tamil-speaking children
	Attended the Ophthalmology Unit of Teaching Hospital Jaffna for eye care during the study period
	Diagnosed with a refractive error
Exclusion	Presence of ocular conditions other than refractive errors that could independently affect vision
	Presence of other significant ocular pathologies requiring specialized intervention
	History of ocular surgery
	Presence of systemic conditions known to impair vision (e.g., corneal pathology or neurological disorders)
	Inability or unwillingness to cooperate with standard visual acuity testing or refraction procedures
	Lack of parental/guardian consent or child assent

Sample size and sampling technique

The sample size was determined using the single-proportion formula, based on the expected prevalence of uncorrected ametropia from the Lucknow hospital-based study (82.2%), with a 95% confidence level and a 5% margin of error [4]. The initial sample size was calculated as 227. Assuming a 10% nonresponse rate, 253 participants were included in the study.

The children's eye clinic at Teaching Hospital Jaffna is held every Saturday, with an average of about 50 children per session. The overall study period extended from April to December 2025, covering all phases from conception to analysis. Within this period, active data collection was conducted over a defined two-month interval. Approximately 400 children were estimated to attend the clinic during the data collection period. Based on the calculated sample size of 253 participants, systematic random sampling was used to ensure a representative sample. At the start of each clinic session, all eligible participants were listed sequentially in order of registration. A random starting point between 1 and k (where k=2) was selected using a random number table. Thereafter, every second eligible participant was recruited from that session's list. This systematic random sampling procedure was repeated across consecutive clinic sessions, with recruitment continuing until the target sample size of 253 participants was achieved.

Variables and data collection

Data were collected prospectively using a structured, interviewer-administered questionnaire and a data extraction form. Each child served as the primary subject for the ophthalmic examination, including assessment of visual acuity and refractive status. Information on sociodemographic characteristics, family history of REs, lifestyle factors (including time spent on outdoor activities and screen exposure), and barriers to spectacle use was obtained primarily from parents or guardians. For children aged 12 years and older with adequate comprehension, self-reporting was accepted where appropriate.

The interviewer-administered questionnaire included demographic information, family history of RE, total time spent on outdoor activities, screen exposure (defined as exposure to computers, television, or mobile devices), and barriers to spectacle use. Outdoor time and screen time were each operationally defined as the self-reported average number of hours per day spent in outdoor activities or using screen-based devices (television, computer, smartphone, or tablet), respectively, as estimated by the parent or guardian or, where applicable, the participant. Responses reflected a general daily average rather than being stratified by weekday and weekend patterns. The questionnaire was developed for the purposes of this study and was not subjected to formal psychometric validation before administration.

The data extraction form included uncorrected distance visual acuity (UDVA), BCVA, and RE pattern. The primary outcome was the pattern of RE. Secondary outcomes included BCVA, lifestyle factors (screen time and outdoor activity), family history of REs, and barriers to spectacle use. Data on visual test outcomes were obtained from participants' clinical records using a structured data extraction form based on routine eye examinations conducted in accordance with the standard procedures described below. No additional procedures were performed specifically for this study.

Visual acuity was assessed using a standard Snellen chart or a K-picture chart, depending on the child's age and cooperation, at a testing distance of 6 m. UDVA and BCVA were recorded. Objective assessment of RE was performed by an optometrist using a tabletop autorefractor, and a slit-lamp examination was performed by an ophthalmologist to assess ocular status and support the evaluation of REs. For cycloplegic refraction, a pre-examination assessment was conducted for all participants to rule out contraindications, including known allergy to cycloplegic agents. This was followed by measurement of intraocular pressure, baseline visual acuity, and pupil size. Cycloplegia was induced by instilling 1% cyclopentolate. A waiting period of 20-45 minutes was allowed to achieve full cycloplegic effect, confirmed by a dilated pupil size ≥6 mm. Once adequate cycloplegia was confirmed, retinoscopy and/or autorefraction were performed through the dilated pupil to determine the objective RE. Following resolution of the cycloplegic effect, postmydriatic manifest refraction was performed to confirm and finalize the refractive correction prescribed for each participant.

Operational definitions of refractive error patterns

REs were classified using spherical equivalent (SE) and cylindrical power, following internationally accepted conventions. Myopia was defined as SE ≤-0.50 D, and hyperopia was defined as SE ≥+0.50 D. Astigmatism was defined as cylinder ≥0.75 D. Astigmatism subtypes were further specified. Myopic astigmatism was defined as an RE with cylinder ≥0.75 D in combination with a myopic spherical component (SE ≤-0.50 D), either in one principal meridian (simple) or both principal meridians (compound). Hyperopic astigmatism was defined as an RE with cylinder ≥0.75 D in combination with a hyperopic spherical component (SE ≥+0.50 D), either in one principal meridian (simple) or both principal meridians (compound) [5,6].

Amblyopia was operationally defined as best-corrected visual acuity (BCVA) worse than 6/9 in one eye despite appropriate refractive correction and in the absence of structural ocular abnormalities [7].

Effective refractive error coverage (eREC) was defined as the proportion of the total study population with a diagnosed RE who were currently and effectively using appropriate spectacle correction at the time of assessment. This indicator captures the full continuum of the RE care cascade, from diagnosis through prescription to actual utilization. eREC was calculated as follows: eREC (%)=(No. of participants currently using appropriate spectacle correction / Total no. of participants with diagnosed RE) ×100 [8].

Data analysis

All collected data underwent rigorous review by the research team before data entry to ensure accuracy and completeness. Data were entered into Microsoft Excel 2007 (Microsoft Corp., Redmond, WA, USA) and analyzed using IBM SPSS Statistics for Windows, Version 25.0 (IBM Corp., Armonk, NY, USA). Continuous variables were summarized as means and standard deviations (SDs). Categorical variables were summarized as frequencies and percentages. For key proportions, including the overall prevalence of amblyopia, eREC, and the distribution of RE subtypes, 95% confidence intervals were calculated using the Wilson score method and computed using WINPEPI (PEPI-for-Windows) software (Abramson JH. WINPEPI updated: Computer programs for epidemiologists, and their teaching potential. Epidemiol Perspect Innov. 2011;8:1).

## Results

A total of 253 participants were enrolled in the study, with ages ranging from 6 to 18 years and a mean age of 11.70±3.58 years. Females accounted for more than half of the participants (138, 54.5%), whereas males accounted for 115 (45.5%). Of the total participants, 84 (33.2%) had a family history of RE, and 169 (66.8%) had no family history of RE (Table 2).

**Table 2:** Sociodemographic characteristics of the study participants (n=253) SD, standard deviation

Characteristics	n (%)
Age with mean (SD)	11.70 (3.58%)
		Sex	
Male	115 (45.5%)
Female	138 (54.5%)
Having a family history of refractive error	
Yes	84 (33.2%)
No	169 (66.8%)

Astigmatism was the most common type of RE, affecting 118 (46.6%, 95% CI: 40.6-52.8%) participants, followed by myopic astigmatism (66, 26.1%; 95% CI: 21.1-31.8%), myopia (58, 22.9%; 95% CI: 18.2-28.5%), hyperopia (6, 2.4%; 95% CI: 1.1-5.1%), and hyperopic astigmatism (5, 2.0%; 95% CI: 0.8-4.5%) (Table 3).

**Table 3:** Refractive error patterns among the study participants (n=253) CI, confidence interval

Refractive error patterns	n (%)	95% CI
Astigmatism	118 (46.6%)	40.6-52.8%
Myopic astigmatism	66 (26.1%)	21.1-31.8%
Myopia	58 (22.9%)	18.2-28.5%
Hyperopia	6 (2.4%)	1.1-5.1%
Hyperopic astigmatism	5 (2.05%)	0.8-4.5%

Analysis of participants by RE pattern showed that the mean age varied across RE categories. Participants with myopia had the highest mean age (13.17±3.26 years), followed by those with myopic astigmatism (12.18±3.45 years) and astigmatism (10.96±3.55 years). Lower mean ages were observed among participants with hyperopic astigmatism (7.60±2.07 years) and hyperopia (10.00±3.29 years). Regarding sex distribution, females were slightly more represented across most RE categories. Among participants with myopic astigmatism, 57.6% (n=38) were female and 42.4% (n=28) were male. Similarly, in the astigmatism group, females comprised 55.1% (n=65), whereas males accounted for 44.9% (n=53). In the hyperopic astigmatism group, 60.0% (n=3) were female, and 40.0% (n=2) were male. In contrast, an equal sex distribution was observed in both the myopia and hyperopia groups, with males and females each accounting for 50.0%. In the myopia group, there were 29 (50.0%) males and 29 (50.0%) females, whereas in the hyperopia group, there were 3 (50.0%) males and 3 (50.0%) females.

A positive family history of REs was reported in approximately one-third of participants across most categories. Specifically, 30.3% (n=20) of those with myopic astigmatism and 31.4% (n=37) of those with astigmatism reported a family history. Higher proportions were observed in the hyperopic astigmatism (n =2, 40.0%), myopia (n=22, 37.9%), and hyperopia (n=3, 50.0%) groups. The majority of participants in all categories reported no family history of REs (Table 4).

**Table 4:** Refractive error patterns by age, sex, and family history among schoolchildren (n=253) SD, standard deviation

Category	Myopic astigmatism	Astigmatism	Hyperopic astigmatism	Myopia	Hyperopia
Age, mean (SD)	12.18 (3.45)	10.96 (3.55)	7.60 (2.07)	13.17 (3.26)	10.00 (3.29)
Sex, n (%)					
Male	28 (42.4%)	53 (44.9%)	2 (40.0%)	29 (50.0%)	3 (50.0%)
Female	38 (57.6%)	65 (55.1%)	3 (60.0%)	29 (50.0%)	3 (50.0%)
Family history of refractive error, n (%)					
Yes	20 (30.3%)	37 (31.4%)	2 (40.0%)	22 (37.9%)	3 (50.0%)
No	46 (69.7%)	81 (68.6%)	3 (60.0%)	36 (62.1%)	3 (50.0%)

The mean outdoor time and screen time varied across RE categories, with generally modest differences between groups. Participants with myopia reported the highest mean outdoor time (2.30±1.51 hours), followed by those with astigmatism (2.00±1.08 hours) and myopic astigmatism (1.94±0.79 hours). Mean outdoor time was lower among participants with hyperopia (1.50±0.84 hours), with the lowest reported in the hyperopic astigmatism group (1.00±0.00 hours).

Regarding screen time, participants with myopic astigmatism had the highest mean duration (1.68±0.92 hours), closely followed by those with astigmatism (1.54±0.90 hours) and myopia (1.47±0.85 hours). Screen time was shorter among participants with hyperopic astigmatism (1.20±0.45 hours) and was lowest among those with hyperopia (0.91±0.66 hours) (Table 5).

**Table 5:** Mean outdoor exposure and screen time across different refractive error patterns (n=253) SD, standard deviation

Category	Myopic astigmatism	Astigmatism	Hyperopic astigmatism	Myopia	Hyperopia
						Outdoor time in hours, mean (SD)	1.94 (0.79)	2.00 (1.08)	1.00 (0.00)	2.30 (1.51)	1.50 (0.84)
Screening time in hours, mean (SD)	1.68 (0.92)	1.54 (0.90)	1.20 (0.45 )	1.47 (0.85)	0.91 (0.66)

**Figure 1:**
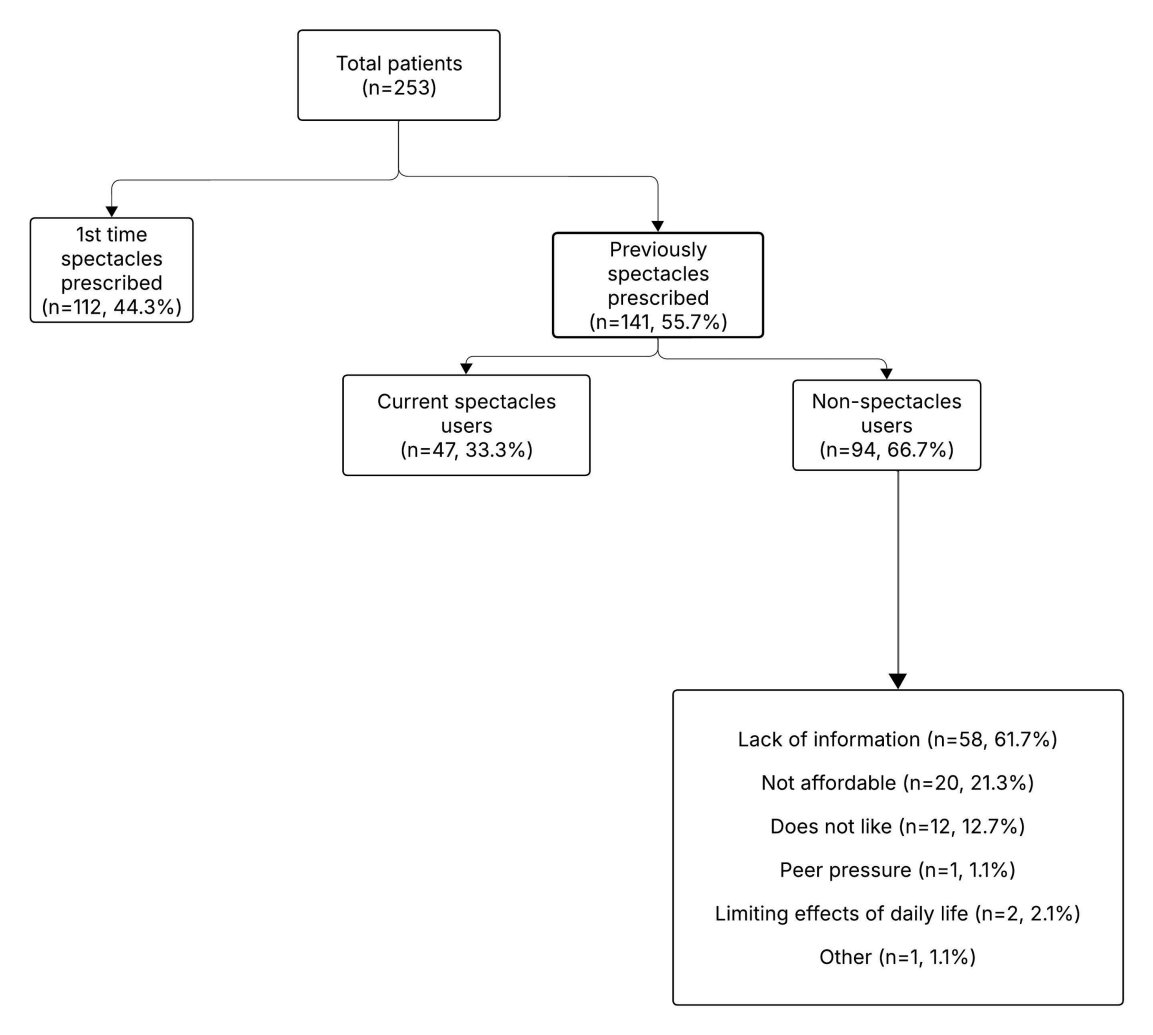
Spectacle prescription status, utilization, and barriers to use among participants (n=253)

The eREC, defined as the proportion of the total population with RE who were currently and effectively using appropriate spectacle correction, was 47 of 253 (18.6%; Wilson 95% CI: 14.3%-23.8%). Of the total study population, 141 (55.7%) had previously been prescribed spectacles, whereas 112 (44.3%) received a first-time prescription during the assessment. Among those with a prior prescription (n=141), only 47 (33.3%) were current spectacle users at the time of the study, whereas 94 (66.7%) were not using their prescribed spectacles. The primary reasons for nonuse of spectacles (n=94, 66.7%) were lack of information (n=58, 61.7%) and affordability (n=20, 21.3%). Other reported reasons included dislike of spectacles (n=12, 12.7%), peer pressure (n=1, 1.1%), and perceived limitations in daily activities (n=2, 2.1%). A small proportion cited other unspecified reasons (n=1, 1.1%) (Figure 1).

Of the 253 participants with diagnosed RE, 152 (60.1%; 95% CI: 53.9%-65.9%) had amblyopia, whereas 101 (39.9%) did not. Among participants with amblyopia, the highest prevalence was observed in hyperopic astigmatism (n=5, 100%) and hyperopia (n=5, 83.3%), followed by astigmatism (n=77, 65.3%) and myopic astigmatism (n=42, 63.6%). Myopia showed the lowest prevalence (n=23, 39.7%) (Table 6).

**Table 6:** Prevalence of amblyopia by refractive error pattern (n=253)

Refractive error pattern	Total no. of participants	Amblyopia present, n (%)	Amblyopia absent, n (%)
Myopic astigmatism	66	42 (63.6%)	24 (36.4%)
Astigmatism	118	77 (65.3%)	41 (34.7%)
Hyperopic astigmatism	5	5 (100.0%)	0 (0.0%)
Myopia	58	23 (39.7%)	35 (60.3%)
Hyperopia	6	5 (83.3%)	1 (16.7%)
Total	253	152 (60.1%)	101 (39.9%)

## Discussion

This study describes the pattern of REs, along with the demographic and behavioral characteristics of the study population, the burden of amblyopia, and eREC among 253 children aged 6-18 years attending the Ophthalmology Unit of Teaching Hospital Jaffna. The mean age of 11.7 years reflects a population in which REs are typically identified during the school years, a critical period for visual development and educational performance. This finding is similar to that of a study conducted at a tertiary eye care center in Western India, where the mean age was 11.01 years [9].

Astigmatism was the predominant RE pattern in this study population (46.6%), followed by myopic astigmatism (26.1%) and myopia (22.9%). This pattern is broadly consistent with findings from a study by Adhikari et al. on RE patterns among children attending Biratnagar Eye Hospital, Nepal, which similarly reported astigmatism as the most common type (35.5%) [10], whereas myopia predominated in other studies, ranging from 19.6% to 84.77% of children with REs [9,11-13]. As both our study and several comparator studies were hospital-based, some of this variation may reflect differences in case-mix and referral pathways, in addition to true regional differences in RE patterns. This issue is discussed further below.

The observed age trend, with a higher mean age among participants with myopia (13.17 years), is consistent with the well-established association between increasing age and progression toward myopic refractive states during adolescence reported in the literature. A study of REs among children in an urban population in New Delhi reported a myopia prevalence of 10.8% among 15-year-olds [14]. Similarly, a higher risk of myopia in older children was of borderline statistical significance (P=0.069) among school-aged children in the rural population of Mahbubnagar District, Andhra Pradesh [15].

Similar to a cross-sectional study conducted in Nepal, where the percentage of REs in females was higher (about 12%) than in males (about 7%) with a P-value of <0.05 (Bhandari et al., 2021), females in the present descriptive study were also slightly more numerically represented across most RE categories, although an equal distribution was observed in the myopia and hyperopia groups. These findings are descriptive observations only and were not subjected to statistical testing because of the small subgroup sample sizes. In contrast, Tiwari et al. (2026) reported a male predominance, with males comprising 53.3% of the study population [13]. While sex differences in RE prevalence are inconsistently reported across studies, this slight female predominance may reflect differences in health-seeking behavior, screening uptake, or true epidemiologic variation.

A positive family history of RE was reported in approximately one-third of participants overall, with a numerically higher proportion in the hyperopia and hyperopic astigmatism groups. As this study did not include a comparison group of children without RE, and family history was not subjected to statistical testing by RE subtype, this finding should be interpreted as a descriptive observation of family history prevalence within an already affected clinical population, rather than as evidence of an association or genetic risk within our sample. Furthermore, the majority of participants in our cohort reported no family history, suggesting that non-genetic and environmental factors likely also play a substantial role, although this study was not designed to formally evaluate their relative contribution.

Behavioral factors such as outdoor activity and screen time showed only modest variation across RE categories in this descriptive analysis. Participants with myopia reported the highest mean outdoor exposure, although differences between groups were small. We note that this study did not include an unaffected comparison group and did not statistically test outdoor time or screen time against RE or amblyopia outcomes; therefore, these findings should not be interpreted as evidence of a protective or risk association within this cohort and, in particular, cannot distinguish a true protective association from reverse causation or unmeasured confounding. This is a relevant distinction given that large-scale, appropriately controlled epidemiologic studies, such as the Sydney Myopia Study, which demonstrated that increased outdoor activity was associated with significantly lower odds of myopia [16], and the Guangzhou Outdoor Activity Study, which found that additional outdoor exposure reduced myopia incidence among schoolchildren using a randomized, objectively measured design [17], used comparison groups and, in some cases, objective exposure measurements to establish these associations, none of which our study design permits. Our descriptive finding that participants with myopia in this cohort reported the highest mean outdoor time should therefore be interpreted only as a description of behavior within an already diagnosed group and is presented as a hypothesis-generating observation consistent with, rather than confirmatory of, the possibility that outdoor activity patterns may differ less once an RE is already established [18]. We did not assess, and therefore cannot comment on, whether outdoor exposure differed before the onset of myopia in this cohort.

A key and well-supported finding of this study is the very low eREC of 18.6% (Wilson 95% CI: 14.3%-23.8%). Although more than half of the participants (55.7%) had previously been prescribed spectacles, only one-third were current users at the time of the study. This finding is similar to that of a cross-sectional study in India, which reported that fewer than one-third of students were compliant with their spectacle prescriptions [19], and is consistent with findings from studies conducted in China [20], Tanzania [21], and Mexico [22]. This indicates a substantial gap between prescription and sustained spectacle use, highlighting that access to diagnosis and prescription alone does not ensure effective correction of RE. The primary barriers identified, lack of information (61.7%) and affordability (21.3%), suggest that both knowledge-related and economic constraints contribute significantly to poor compliance. These findings are consistent with reports from other studies, in which nonuse of spectacles is commonly driven by misconceptions about need, stigma, and cost-related barriers rather than by prescription availability alone [21,22].

Similarly, the 60.1% prevalence of amblyopia among children with RE is notably high and raises important public health concerns. Amblyopia was particularly prevalent in hyperopic astigmatism (100%) and hyperopia (83.3%), consistent with the established understanding that uncorrected hyperopic REs in early childhood carry a higher risk of amblyopia because of sustained retinal defocus. However, the very small sample sizes in the hyperopia (n=6) and hyperopic astigmatism (n=5) groups limit the reliability of these percentage estimates, underscoring the need for larger, multicenter studies to validate these findings. Data from the Multi-Ethnic Pediatric Eye Disease Study and the Baltimore Pediatric Eye Disease Study show that hyperopia ≥4.00 D is associated with bilaterally reduced visual acuity (OR 10.8), and hyperopia severity is associated with esotropia risk ranging from OR 6.4 (2.00-<3.00 D) to OR 122.0 (≥5.00 D) [23-25]. Similarly, astigmatism shows a severity-dependent association with reduced visual acuity, with odds ratios increasing from 2.3 (1.00-<2.00 D) to 17.6 (≥2.00 D) [26]. The Vision in Preschoolers Study also demonstrated a dose-response relationship between bilateral astigmatism and bilateral amblyopia risk, with odds ratios increasing from 3.3 (1-2 D) to 20.9 (3-4 D). The relatively low prevalence of myopia (39.7%) further supports the differential amblyogenic potential of RE subtypes.

The present study, with a sample of 253 participants, offers valuable preliminary insights but has several limitations. Although adequate for descriptive reporting, the moderate sample size limits statistical power and precision, particularly for less common RE subtypes such as hyperopia (n=6) and hyperopic astigmatism (n=5). This may exaggerate percentage values and restrict generalizability. The reliance on descriptive analysis without inferential or multivariate methods limits the ability to adjust for confounding factors such as socioeconomic status, family history, or educational exposure. Additionally, because this is a cross-sectional study, it captures the prevalence of RE and amblyopia at a single point in time. It cannot establish causality or temporal progression, particularly regarding age-related changes or behavioral factors such as outdoor exposure. Furthermore, reliance on self-reported behavioral data (e.g., outdoor exposure and screen time) introduces recall bias.

A further limitation relates to selection bias inherent to the hospital-based, tertiary-referral design of this study. Participants were drawn exclusively from children who were referred to, or who self-presented at, a tertiary eye care center and had already been diagnosed with an RE before or at the time of enrollment. This referral pathway is likely to enrich the study sample with more severe, symptomatic, or complex ocular disease compared with an unselected community- or school-based population, in which milder or asymptomatic cases would also be captured through routine screening. Consequently, several of our findings, including astigmatism predominance, amblyopia prevalence, and sex distribution, that have been benchmarked against community- and school-based surveys from other countries should be interpreted with caution, as a tertiary referral population is not directly comparable to an unselected school- or community-based population. Observed differences may reflect differences in underlying case severity and referral pathways rather than true differences in disease epidemiology between populations.

In addition, this study's amblyopia prevalence (60.1%) is higher than population estimates (1%-5%) [25,26] because it was conducted at a tertiary hospital eye unit, which receives referrals for complex cases, thereby enriching the sample with significant ocular pathology, including amblyopia. It included only children with diagnosed REs, a known risk factor for amblyopia, thereby increasing the observed prevalence. Delayed detection, inconsistent spectacle use, and the lack of routine preschool screening may also have contributed, as children aged 6-18 years may develop amblyopia before diagnosis. These findings reflect the burden of amblyopia among children with REs attending a tertiary center, rather than in the general population. Although they highlight the importance of early screening, population-based studies are needed to determine the true prevalence and inform screening policies.

Despite these limitations, this study represents, to our knowledge, the first hospital-based study of RE and amblyopia conducted in Sri Lanka and specifically addresses a notable gap in hospital-based, as opposed to school-based, evidence from Northern Sri Lanka. As such, it provides a valuable clinical population reference point that complements, rather than replaces, community-based epidemiologic data from this region.

Overall, these findings reveal two key gaps in the management of REs: the need for early detection and consistent correction. Although initial spectacle prescriptions are generally adequate, challenges such as poor adherence and limited effective coverage reduce the benefits of refractive services. This underscores the need to enhance school-based vision screening, strengthen health education for children and parents, and implement strategies to overcome cost and social barriers to spectacle use.

## Conclusions

This study provides a descriptive account of the pattern of REs, demographic and behavioral characteristics, amblyopia burden, and effective RE coverage among children attending a tertiary hospital eye unit in Northern Sri Lanka. Astigmatism was the most prevalent RE, followed by myopic astigmatism and myopia. Hyperopia and hyperopic astigmatism were less common but were associated with the highest prevalence of amblyopia, despite the very small sample sizes. The overall high prevalence of amblyopia indicates a significant public health concern. Females were numerically more represented across most RE categories, although this difference was not statistically significant, and about one-third of the children reported a family history of RE. As this study was descriptive in design and did not include a comparison group or inferential statistical testing for these variables, these findings should be regarded as observations within this population rather than as evidence of an association. Variations in outdoor activity and screen time were modest across groups; children with myopia spent the most time outdoors, whereas those with myopic astigmatism reported the longest screen time.

Effective RE coverage was particularly low (18.6%), falling well below international benchmarks, with many participants not wearing their prescribed spectacles because of limited awareness and affordability challenges. This finding, together with the high amblyopia burden, represents the most robust and clinically actionable outcome of this study and underscores the urgent need for early vision screening, improved access to affordable corrective services, and ongoing follow-up to prevent long-term visual disability. Larger, population-based, and analytically designed multicenter studies are needed to confirm these observations, formally test the associations suggested by the broader literature, and inform national strategies for childhood eye health in Sri Lanka.
